# Exploring the Influence of a Novel App for Training and Evaluating Walking Aid Skills in Walking Aid Users: Protocol for a Pragmatic Single-Blind Randomized Controlled Trial

**DOI:** 10.2196/71060

**Published:** 2025-12-24

**Authors:** Félix Nindorera, Ranita H K Manocha, William C Miller, François Routhier, Krista L Best

**Affiliations:** 1 Faculty of Medicine School of Rehabilitation Sciences Université Laval Québec, QC Canada; 2 Centre for Interdisciplinary Research in Rehabilitation and Social Integration Centre intégré universitaire de santé et de services sociaux de la Capitale-Nationale Québec, QC Canada; 3 Department of Clinical Neurosciences University of Calgary Calgary, AB Canada; 4 Faculty of Kinesiology University of Calgary Calgary, AB Canada; 5 Department of Occupational Science and Occupational Therapy University of British Columbia Vancouver, BC Canada; 6 Centre for Aging SMART Rehabilitation Research Program GF Strong Rehabilitation Centre Vancouver, BC Canada

**Keywords:** walking aids, mobile health, mHealth, balance confidence, mobility, randomized controlled trial

## Abstract

**Background:**

More than 12% of the world’s population and more than 1 million Canadians use walking aids to support mobility. Unsafe use of walking aids due to a lack of training may lead to injuries and an increased risk of falls. A novel interactive video-based feedback mobile app to train walking aid fitting and safe use, called ICanWALK (Improving Canadians’ Walking Aid Skills, Learning, and Knowledge), was recently developed.

**Objective:**

The primary objective of this study is to explore the efficacy of the walking aid skills training app on the balance confidence of walking aid users. The secondary objective is to explore the influence of the mobile app on mobility and knowledge of walking aid users.

**Methods:**

A 2-site single-blind pragmatic randomized controlled trial is proposed. A total of 52 adults who use walking aids will be recruited through clinical and community organizations. Participants will complete measures of balance confidence (Activities-specific Balance Confidence scale; the primary outcome), mobility (6-Minute Walk Test and Timed Up and Go test), walking aid skills (Walking Aids Skills Test, WAST), and knowledge of and confidence in walking aid fit and use (self-reported questionnaire) at baseline (T1). Participants will then be randomly assigned to the experimental (ICanWALK app) or attention-matched control group (breathing activity app) and will complete two 20-minute sessions interacting with the assigned app. Participants will be reassessed 2 to 4 days after the intervention (T2) and again 4 weeks later (T3). Analysis of covariance will be performed for primary and secondary outcomes by using SPSS software.

**Results:**

The study protocols were approved by the institutional review boards of both recruitment sites in 2023 and 2024. A feasibility study was conducted from 2023 to 2024 across the 2 sites. As of November 2025, participant recruitment is ongoing and expected to conclude in December 2026. To date, 26 individuals have been enrolled and have successfully completed all 3 assessment time points: T1, T2, and T3.

**Conclusions:**

Using a video-based feedback training approach, a novel app is hypothesized to improve balance confidence, mobility, and knowledge of and confidence in walking aid fitting and use. This structured educational program for fitting and training of walking aids may improve balance confidence. Better walking aid fitting and use may improve mobility, especially for older adults, thereby increasing independence and social participation. Establishing efficacy is an important first step before exploring how the ICanWALK app may be used by walking aid users and clinicians in clinical and community settings.

**Trial Registration:**

ClinicalTrials.gov NCT05347875; https://clinicaltrials.gov/study/NCT05347875

**International Registered Report Identifier (IRRID):**

DERR1-10.2196/71060

## Introduction

Independent personal mobility is associated with physical and psychosocial health benefits, such as reduced risk of chronic diseases, increased participation in meaningful activities, and reduced risk of depression and social isolation [1,2]. Personal mobility, defined as the ability to move oneself (either independently or using assistive devices) within environments [3,4], is a basic human right [3]. According to the Convention on the Rights of People with Disabilities, state parties shall take effective measures to ensure personal mobility with the greatest possible independence [3], which includes facilitating access to quality mobility aids and providing mobility skills training.

The World Health Organization (WHO) and World Bank estimated that 1.3 billion people (representing approximately 15% of the world’s population) experience significant disability, with most of them using walking aids, designed to assist ambulation, such as canes, walkers, crutches, and walking sticks [5,6]. In Canada, walking aids support the independent mobility of more than 1 million Canadians (approximately 4.1% of the population), with a 2% prevalence increase from 2004 to 2012 [7]. Walking aid use is particularly prevalent among older adults [7,8], with approximately 24% of adults aged 65 years and older in the United States and Canada using walking aids [7,8]. With life expectancy rising worldwide, the population older than 65 years is expected to continue to increase [9], and, consequently, the need for walking aids will also increase [10].

When used appropriately, walking aids can significantly enhance perceived balance confidence in individuals with mobility challenges, especially older adults and those with neurological conditions [11]. Improved balance confidence has been shown to facilitate motor performance, leading to smoother and more efficient movements [12,13]. Walking aids support postural stability, compensate for muscle weakness or motor deficits, and off-load weight from an injured or postoperative lower limb [12]. Their use is associated with greater feelings of safety, which may contribute to a higher level of physical activity, independence, and reduced risk of fall-related injuries [13,14]. Overall, walking aids support mobility [15], encourage participation [11], and offer both physical and psychosocial benefits [16].

Notwithstanding the numerous benefits of walking aids, inappropriate fit and unsafe use can lead to discomfort, pain, injuries, and even an increased risk of falls. A recent review identified an increased risk of injury when crutches were poorly fitted and used improperly due to a lack of training before use [17]. Inadequate use of walking aids was also found to be associated with a greater risk of falls [15,18]. In US emergency departments, 47,312 fall-related injuries among older adults, which were associated with walking aids (87.3% with walkers, 12.3% with canes, and 0.4% with both), were treated annually between 2001 and 2006 [19]. Furthermore, high rates of walking aid disuse or dissatisfaction were associated with difficult or risky use due to a lack of training before prescription [20]. Although injuries and fall risks could likely be reduced through better education and training, no standardized training program has been documented. A recent study by Best et al [21] that documented the quality of existing online educational resources for mobility device training showed that most walking aid training was in the form of infographics and basic instructions and was not evidence based. Most people obtain walking aids independently from the pharmacies or clinics without receiving instruction or demonstration [20,22]. A standardized and effective approach to training walking aid skills may enhance balance confidence and mobility while reducing the risk of injuries and falls for walking aid users and may be useful for health care professionals (as they are the primary source of care for patients).

The use of mobile apps is increasingly widespread and recognized for improving health outcomes in the general population [23] as well as in individuals with chronic diseases [24]. The COVID-19 pandemic also facilitated the use of mobile health (mHealth) for providing some health services and reducing initial preoccupations about safety, which has increased accessibility to health care services and education while reducing geographic and transportation barriers [24]. While educational mHealth apps have demonstrated potential in enhancing health-related knowledge and promoting behavior change [25-28], their overall effectiveness remains inconsistent. This variability is largely attributable to differences in the integration of key design and theoretical elements, such as behavior change techniques, personalization, user-centered design, social support features, evidence-based content, and technical reliability [29-32]. In the specific context of rehabilitation, some apps incorporate video-based instructional content; however, they typically offer passive viewing experiences and lack interactive features that support active skill acquisition [25,33-35]. Critically, few enable users to engage in guided practice with real-time or postexercise feedback that facilitates the detection and correction of performance errors by comparing their movements to a model [36]. Moreover, the use of video-based feedback, an evidence-informed strategy grounded in observational learning theory, remains notably underused, despite its potential to enhance motor skill acquisition, user engagement, and self-efficacy [37,38]. In addition, no educational app for teaching the fitting and safe use of walking aids exists to date.

The ICanWALK (Improving Canadians’ Walking Aid Skills, Learning, and Knowledge) mobile app was developed to address critical gaps related to walking aids, including improper fitting, unsafe use, and the widespread lack of structured training [39]. The ICanWALK app is a video-based feedback and interactive tool aiming to serve as a large-scale educational tool [39]. A recent pilot experimental study involving 13 walking aid users reported a high rate of usability (88%) and acceptability of this novel app [40]. Participants found the app simple, useful, and easy to use [40]. This study also showed improvement in balance confidence after two 15-minute sessions using the ICanWALK program [40]. Hence, this study will explore the efficacy of the ICanWALK program on the primary outcome of balance confidence in adult walking aid users. We hypothesize that exposure to the app’s content (video-based feedback and interactive learning) will lead to improved balance confidence (main variable), and we expect that if balance confidence improves, mobility and walking aid skills (secondary outcomes) will also improve.

## Methods

### Study Design

This study will use an assessor-blind, 2-site parallel-group, randomized controlled trial (RCT). This study protocol was registered on ClinicalTrials.gov (NCT05347875). The SPIRIT (Standard Protocol Items Recommendations for Intervention Trials) guidelines [41] and the CONSORT (Consolidated Standards of Reporting Trials) statement [42] will be followed.

### Participants and Recruitment

**Figure 1:**
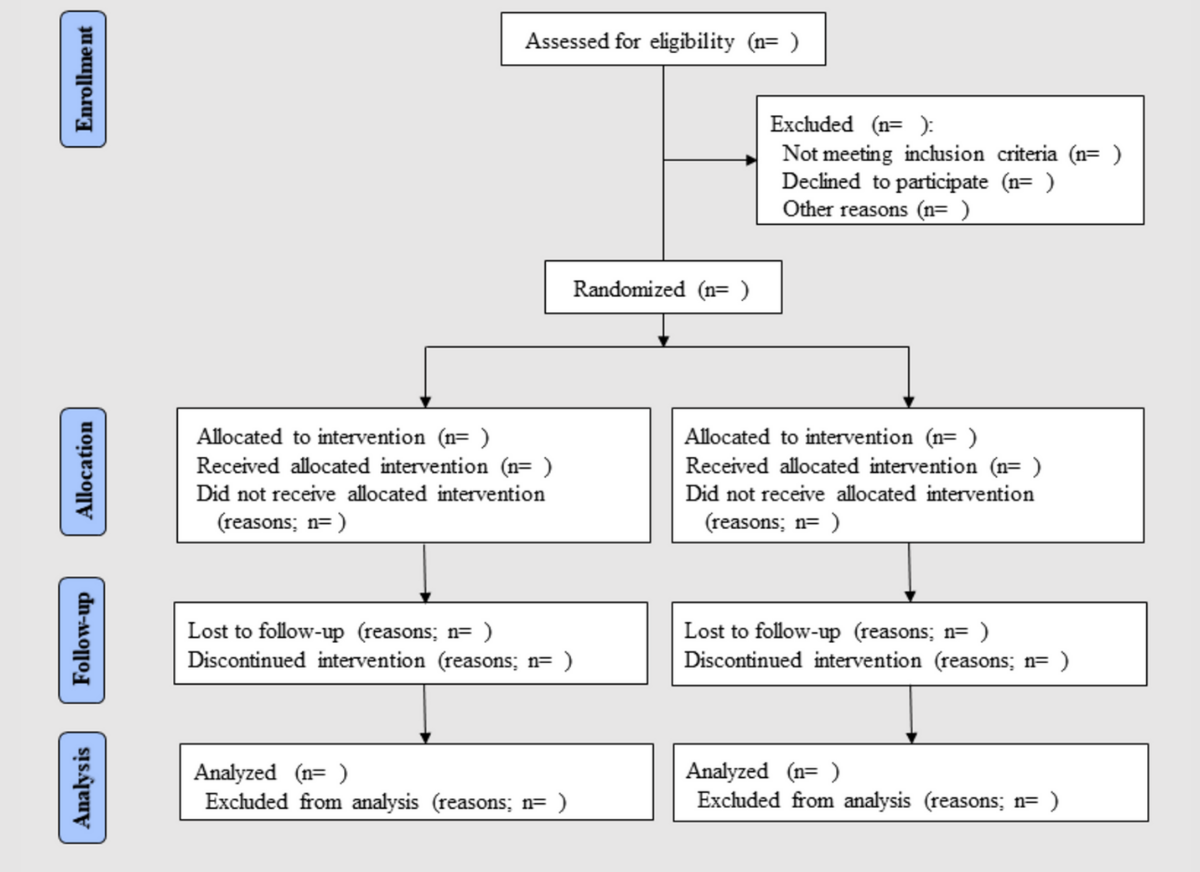
Flow diagram of the progress through the phases of a parallel randomized controlled trial of 2 groups.

To be included in this study, participants should be aged 18 years or older, should use a walking aid (cane, crutch, or walker) for at least 75% of ambulatory time (self-reported), and should be able to communicate in English or French. Participants will be excluded if they have a progressive condition or injury that may restrict or be worsened by walking aid use (self-reported) or a visual impairment that could limit their ability to interact with a mobile app (self-reported). Reasons for exclusion, declining participation, and dropout will be documented for CONSORT purposes (Figure 1) [33,42].

Participants will be recruited from clinics, community centers, semiprivate assisted living facilities, and via social media and emails distributed to health care practitioners by the study coordinators (FN and RHKM). Recruitment posters will be distributed, and emails will be sent to community associations and organizations, practitioners (kinesiologists, physiotherapists, and occupational therapists) in contact with walking aid users, and all university and research center networks. Active approaches, such as community meetings with presentations and collaboration with community partners in identifying potential participants, will also be used. The study coordinators from the 2 sites (FN for Québec and RHKM for Calgary) will contact interested, eligible participants via email and telephone and send them a consent form at least 24 hours before the first evaluation.

### Sample Size

The sample size calculation was based on the Activities-specific Balance Confidence (ABC) scale questionnaire (primary outcome) [43]. No study has reported the minimum clinically important difference for the ABC scale among walking aid users. However, considering a study on 376 patients with knee osteoarthritis who had undergone surgery (many of whom used a walking aid), ABC scores increased by an average of 9.2% 1 year after total knee arthroplasty (repeated measures model; Cohen *d*=0.59) [44]. On the basis of the variability reported in the study [44], an estimated correlation of 0.5 between measurements, including ABC scale and 2 potential covariates (type of walking aid and duration of walking aid use), a sample size of 45 would be sufficient to explore a 2-way difference between groups (*F* tests) in a 2-way repeated measures model (2 groups×2 time points; 1–β=.9; α=.05; G*Power [Heinrich Heine University Düsseldorf]) [45]. With a conservative dropout rate estimated at 15% [46], a total sample size of 52 participants is enough to find significant differences between groups for the primary outcome. To minimize participant dropout and ensure high retention rates, several strategies will be implemented. These include clear and transparent informed consent procedures, regular and supportive communication with participants, and flexible scheduling for study visits. Efforts will be taken to maintain a strong and supportive relationship between the research team and participants. All participants will be reassured about the confidentiality of their data and the importance of their contribution to the success of this study.

### Randomization and Allocation

#### Overview

The randomization will be performed by a researcher not directly involved in this study using a computer-generated program. Participants will be randomly assigned to either the experimental group (the ICanWALK group) or the control group (the breathing exercise group) in a 1:1 ratio. Allocation will be performed by the same researcher using opaque envelope assignments.

#### Experimental Group

**Figure 2:**
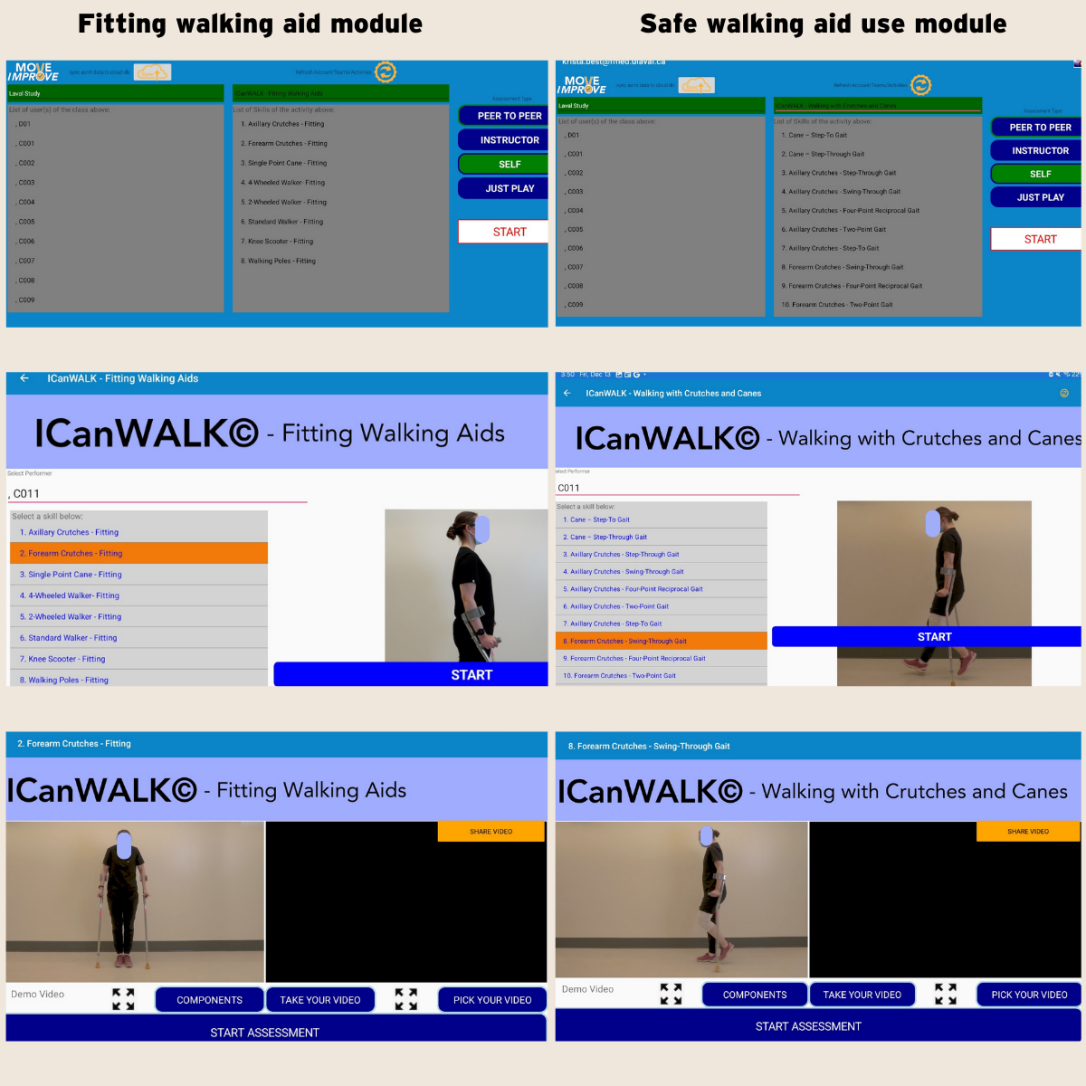
Screenshot of the main steps in using the ICanWALK (Improving Canadians’ Walking Aid Skills, Learning, and Knowledge) app modules.

The ICanWALK app was recently developed as an educational tool designed to train skills related to fitting and using a walking aid. The experimental group will use the ICanWALK app on an iPad (Apple Inc), available on the MOVE Improve mobile app for both Android and iOS. The MOVE Improve platform is a valid app designed to teach physical movement and improve sports performance [47-49]. The MOVE Improve is a video-based feedback and interactive tool aiming to serve as a large-scale educational tool. The ICanWALK modules [39] follow the MOVE Improve instructional format. Users select a specific skill and then watch an instructional video on that skill with the key skill components highlighted. The user is then videotaped while performing the skill, and that video is shown beside the instructional video. Next, the user is guided through a series of skill performance questions to evaluate their video. At the end of the module, the user receives a summary assessment score from their self-evaluation. The ICanWALK program currently contains 43 modules that teach walking aid-related skills, including fitting, gait patterns, and navigating stairs [39]. Health science students or research assistants will receive 4 hours of training on navigating MOVE Improve, guiding participants in selecting ICanWALK modules, and recording videos. With assistance from a trained health professional student, participants will complete 2 separate 15- to 20-minute sessions within a 1-week period. Using the ICanWALK program, the participant will learn skills for fitting and using a walking aid. Participants will watch an instructional video on each skill, with the key components of appropriate skill execution highlighted (Figure 2). The participant will then perform the task with their walking aid, and the student/research assistant will record the video. The participant will review the video in the app and compare it to the demonstration video to determine whether each component of the skill was performed correctly. A score will be generated at the end. Participants must master the use of the app to be able to independently repeat the tasks of fitting and using their walking aid.

#### Control Group

**Figure 3:**
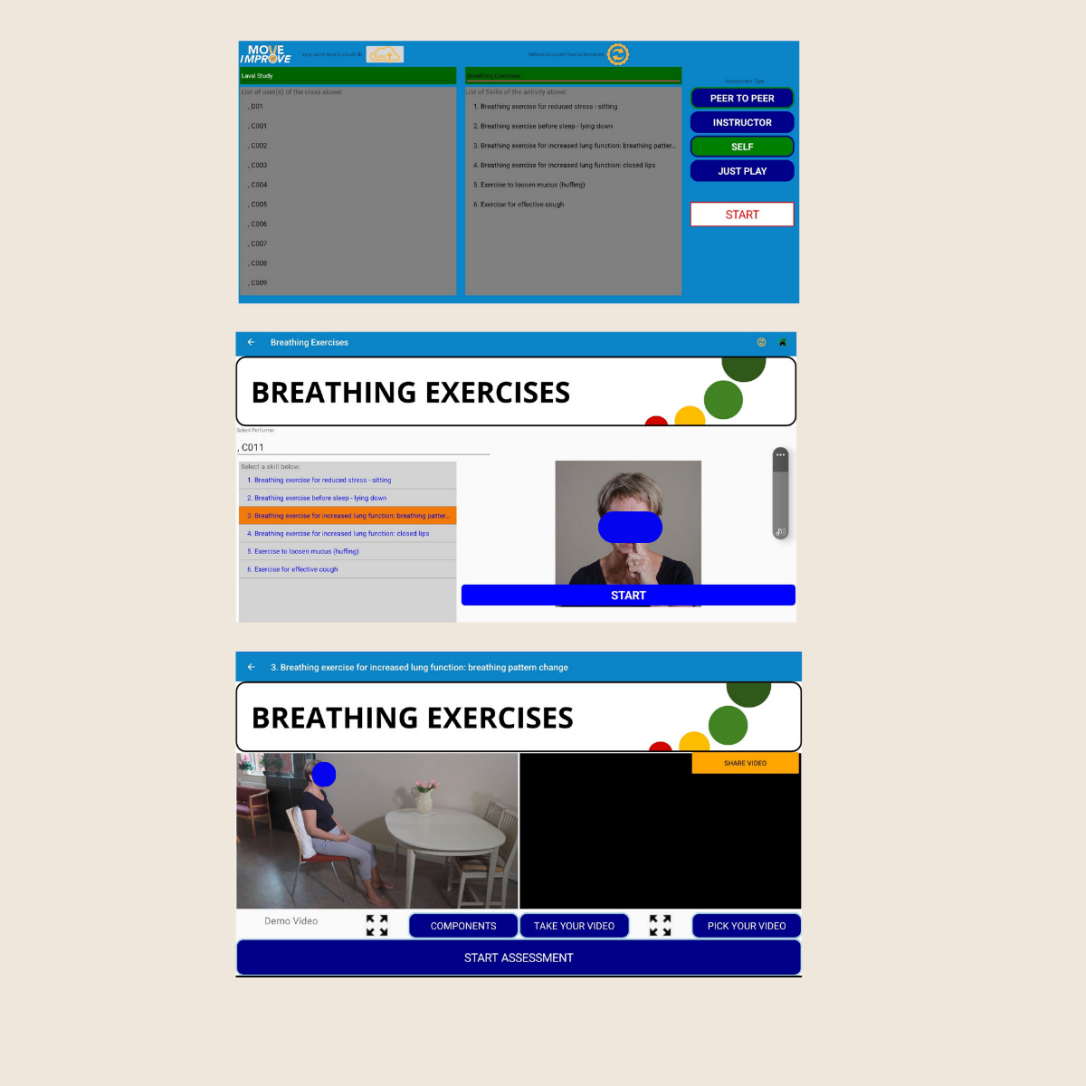
Screenshot of the main steps in using the breathing exercises module.

The control group will be attention matched to mimic the time, location, and contact with a trainer of the experimental group [50]. Participants will follow the same schedule as the experimental group but will also complete a breathing exercise module available on the MOVE Improve app. The modules are identical in layout, instruction, and self-assessment of videos to the ICanWALK app but differ in content, as no information related to walking aids and mobility is included in this program (Figure 3). With assistance from a trained health professional student or a research assistant, participants in the control group will complete 2 separate 15- to 20-minute sessions within 1 week. Participants will select the breathing exercise module and then choose the specific skill they wish to practice (eg, breathing exercise for reduced stress and breathing exercise for increased lung function), watch the instructional video, and review the performance criteria. A video of the participant performing the selected skill is then recorded and uploaded alongside the model video to allow for side-by-side comparison. The participant will review the video in the app and compare it to the demonstration video to determine whether each component of the skill was performed correctly. A score will be generated at the end.

### Study Procedure and Data Collection

The study procedures (timeline and measurement tools) are illustrated in the SPIRIT checklist (Table 1). Participants will be assessed at 3 time points: baseline (T1), after intervention (T2), and 4-week follow-up (T3). After signing the consent form, a study coordinator will schedule the first evaluation session (T1). Preintervention questionnaires will include sociodemographic data (eg, age, sex, gender, walking aid type, reasons for using the walking aid, the duration of walking aid use, and previous walking aid experience), the ABC questionnaire, and self-reported knowledge and confidence in fitting and using the walking aid using a tablet or laptop. Participants will then complete the Walking Aids Skills Test (WAST) and mobility tests (6-Minute Walk Test, 6MWT and Timed Up and Go, TUG test).

**Table 1:** SPIRIT (Standard Protocol Items Recommendations for Intervention Trials) checklist.

Schedule of activities		Enrollment	Assessment	Allocation	After allocation				Closeout
		Screening	Baseline (T1)		Intervention	After intervention (T2)	4-wk follow-up (T3)		
**Enrollment**									
	Eligibility screen	✓							
	Informed consent	✓							
	Allocation			✓					
**Intervention**									
	ICanWALK^a^ app (experimental intervention)				✓				
	Breathing exercises app (control intervention)				✓				
**Assessments**									
	Activities-specific Balance Confidence scale		✓			✓	✓		
	Self-reported walking aid knowledge and confidence		✓			✓	✓		
	Timed Up and Go test		✓			✓	✓		
	6-Minute Walk Test		✓			✓	✓		
	Walking Aid Skills Test		✓			✓	✓		

^a^ICanWALK: Improving Canadians’ Walking Aid Skills, Learning, and Knowledge.

All data will be collected by an assessor who will be blinded to group allocation. The T1, postintervention (T2), and follow-up (T3) questionnaires will be imported into the LimeSurvey platform to allow for completion during the evaluation while ensuring secure data management. The collected data will be anonymized with a code assigned to each participant and will be stored on the institutional platform. Access to data will be limited to authorized personnel.

### Outcomes

#### Primary Outcome

Given the ICanWALK app’s theoretical grounding in self-efficacy, behavior change, and skill acquisition, balance confidence—assessed with the ABC scale—was selected as the primary outcome due to its direct relevance to the intervention’s mechanisms of action [32,43]. The ABC scale offers a validated, context-specific measure of individuals’ perceived confidence in performing daily mobility tasks with a walking aid, capturing both psychological and functional dimensions of change [43]. As such, it serves as a sensitive and theoretically congruent indicator of the intervention’s anticipated impact. The ABC scale is a 15-item self-assessment measure that asks individuals to rate their confidence in balance while performing a series of activities on a scale ranging from 0 (no confidence) to 45 (total confidence) [43]. The raw scores are converted into percentages by dividing the sum of the individual scores by the total score and then scores ranging from 0 (no confidence) to 100% (total confidence). The ABC scale is reliable in individuals using mobility devices [51], healthy older adult community dwellers [52], individuals who have undergone lower limb amputation [51,53], individuals with Parkinson disease [54] or knee osteoarthritis [55], stroke survivors [56], and those with traumatic brain injury [57]. The ABC scale is highly sensitive to fall risk [58]. Previous studies have demonstrated high test-retest reliability (intraclass correlation [ICC]=0.85) and excellent construct validity (*r*=0.60-0.75) with the Berg Balance Scale, Community Balance and Mobility Scale, and TUG test [52,55,56]. The administration time is 10 to 15 minutes.

#### Secondary Outcomes

##### Mobility

Mobility tests will include the 6MWT and the TUG test. The 6MWT assesses walking endurance by objectively measuring the distance covered by an individual during 6 minutes of walking [59,60]. It is a common, valid, and reliable measure of submaximal aerobic capacity in individuals with various conditions that takes about 10 minutes to administer [61]. The TUG test assesses functional mobility and fall risk by recording the time (in seconds) it takes for a participant to stand up, walk 3 m, and sit back down on a chair [62]. Measurement properties of the TUG test have been reported for older adults (test-retest reliability; ICC=0.75) and in patients with hip osteoarthritis (interrater reliability; ICC=0.87) [63,64]. The TUG test takes 1 to 2 minutes to administer.

##### Knowledge and Confidence in Fitting and Using Walking Aids

Subjective self-reported knowledge and confidence in walking aid fitting and using will be documented at T1, T2, and T3 using a 4-level Likert scale ranging from “not at all informed or not at all confident,” meaning any knowledge and confidence, to “well informed or very confident,” meaning high knowledge or good confidence.

##### Walking Aid Skills

The WAST test will be used to objectively assess skills of using walking aid when performing daily life activities (eg, walking on flat solid ground, walking on foam surfaces, and going up or down steep or gentle slope or stairs). The WAST is a 15-item objective assessment that measures an individual’s ability to use their walking aids in various contexts, such as picking up an object from the ground and walking on various grounds such as gravel or slopes. Using a standardized rating system (videos), the evaluator assigns a score from 0 (the participant does not meet most evaluation criteria, is unsafe, or is unwilling to perform) to 3 (the participant performs the skill very competently or advanced). The total score expressed as a percentage (indicating the individual’s performance) is the quotient of the total sum of individual scores over the number of skills performed, ranging from 0% to 100%. The higher the score, the better the performance. Although the psychometric properties of this test have not yet been investigated and published, it has been developed with inspiration from the wheelchair skills program [65,66] but has not yet been validated.

### Data Monitoring

The intervention used in this study is an educational, nonpharmacological approach that adheres to the ethical principles of beneficence and nonmaleficence. Given its low-risk nature, it is unlikely to cause adverse effects. Therefore, the establishment of a data monitoring safety board may not be necessary. However, any adverse effects will be monitored informally by the project coordinators.

### Statistical Analysis

Responses to questionnaires and test scores will be imported from the LimeSurvey platform and compiled into a Microsoft Excel file. Descriptive statistics (means, SDs, frequencies, and percentages) will be used to summarize sociodemographic data.

An analysis of covariance will be performed to detect postintervention differences between groups for the primary and secondary measures using the respective T1 score as the covariate. Dropout rates and reasons will be reported. Depending on the nature of the missing data (proportion of missing values), appropriate methods will be chosen with careful consideration of the data structure and the underlying assumptions. The significance level will be set at .05, and all statistical tests will be 2-tailed. The scores from the self-reported questionnaires on walking aid knowledge and confidence will be summed and analyzed using an appropriate parametric test (if normally distributed). Retention of balance confidence, mobility, and self-reported score of knowledge in the experimental group will be evaluated using 2-tailed paired *t* tests. Sensitivity analyses will also be conducted based on walking aid type, walking aid use experience, previous training, and the main reason for use. All analyses will be performed in SPSS software (version 27; IBM Corp).

### Ethical Considerations

This study protocol has been approved by each of the 2 sites’ board committees: the Centre Intégré Universitaire de Santé et des Services Sociaux of the Capitale Nationale Rehabilitation Sectoral Ethics Committee, Québec (approved on June 10, 2024; 2025-3085) and the University of Calgary Conjoint Health Research Ethics Board in Alberta (REB25-1290). Written informed consent will be obtained from the potential participants. Any modification to the protocol will be reviewed and approved by the corresponding ethics boards and will be reported to all investigators during the team’s monthly meetings.

During the study, the information about participants will be kept confidential, and signed informed consent will be obtained after the participants agree to participate in this study. The whole procedure will strictly follow the principles of voluntariness, confidentiality, and no harm. Participation is entirely voluntary, and participants may withdraw from the study at any time without providing a reason. Withdrawal will not affect access to health services, medical treatment, or any other rights to which participants are entitled.

To protect participant privacy, all data will be stored on secure, password-protected institutional servers and managed within the institution’s Microsoft Teams environment, which provides encrypted, access-controlled data storage. Access to study folders on Microsoft Teams will be restricted to authorized members of the research team who have completed the required institutional privacy and security training. Identifiable information will be stored in a separate, access-restricted location and will not be linked to research data except through encrypted identification codes. All datasets used for analysis will be fully deidentified, and no personally identifying information will appear in any presentations, publications, or knowledge-translation materials. Data storage, retention, and destruction will follow established institutional policies and provincial regulatory requirements.

To minimize participation burden, participants will receive financial compensation of CAD $25 (US $32.50) for each assessment session and will be provided with a parking-lot pass for every in-person visit.

## Results

As of November 2025, 26 participants have been recruited and have completed all 3 assessments: T1, T2, and T3. Participant recruitment began in July 2024 and is expected to conclude by the end of December 2026. The study is projected to be completed by June 2027.

## Discussion

### Anticipated Findings

Through this single-blind, 2-site RCT, we aim to evaluate the primary hypothesis that an education and training tool for the correct use of walking aids will effectively improve balance confidence and secondarily may influence mobility and walking aid skills. This study protocol should allow us to determine whether the novel video-based feedback educational tool, designed to teach and train fitting and safe use of walking aid, is effective in improving balance confidence, mobility, walking skills, and knowledge. This is an exploratory RCT, given the paucity of literature on the effectiveness of mobile educational apps on short- and long-term walking aid use.

To meet the challenges of the lack of formal training programs for users on the fitting and use of walking aids and the lack of standardized information for health care professionals, mHealth apps may constitute an affordable and scalable digital health intervention to address these growing gaps. The findings from this study will provide valuable insights into the effectiveness of this novel app in improving knowledge of walking aid fitting and safe use as well as improving balance confidence and mobility. Recent evidence suggests that physiotherapy interventions delivered through a smartphone or tablet app were effective in improving mobility outcomes in people with a wide range of health conditions (such as neurological disorders [67,68]). The video-based feedback learning method used by the ICanWALK modules is known for rapidly improving memory recall [69], behavior change [70], and motor skill acquisition [71] and, consequently, should prevent falls while improving functional independence.

The findings of this study may help improve accessibility to health care by reducing the waiting time between the walking aid prescription, purchase, and training with a health care professional. In fact, while some walking aids are owned independently, others are prescribed by health care professionals, with physical therapists being the most common prescribers [72]. Thus, this app would contribute to reducing the waiting time for walking aid prescriptions and training with a health care professional. For instance, more than 10,000 Canadians were reported to be waiting for occupational therapist or physiotherapist services across Ontario [73], and the median waiting time was more than 6 months for 41% of outpatient publicly funded physiotherapy services in Québec [74]. Improving access to mHealth technologies may help address the growing human health care resource challenges in the world [75] and may also enable access to education for people living in remote or rural areas.

Finally, the results obtained from this pragmatic RCT, especially from sensitivity analysis, may help identify specific factors contributing to improved balance confidence, mobility, and knowledge of walking aid users after using an educational tool, such as the type of walking aid, the previous experience using walking aid, and individual factors such as age and diagnosis. This will enable larger-scale studies to be set up by the type of walking aid or the type of condition necessitating the use of a walking aid.

### Limitations

This study may have several limitations. First, we will include participants who use various types of walking aid, but we will not stratify them according to type of walking aid, their digital literacy, cognitive level, user experience, or education. This may result in inconsistent responses to the app. However, sensitivity analyses will help to partially address this issue. Furthermore, participants with cognitive impairments or vision or hearing limitations may face barriers to fully engaging with the app. Second, the primary outcome is a self-reported measure, and this may be subject to measurement bias, such as social desirability bias or recall bias. We believe that the study design, methodology, and expected sample size make the expected results plausible. Finally, this study focuses on a short-term follow-up period and may not capture the long-term efficacy of the app. The key strength of this study will be the use of video-based feedback, as it enables the motor learning process and skills acquisition, leading to high memorization, behavioral change, and performance [69,71,76,77].

### Comparison With Prior Work

To our knowledge, this study will be the first to investigate the effectiveness of an educational tool that teaches and trains the fitting and use of walking aids.

## Data Availability

This is a study protocol; no datasets were generated or analyzed during this study. Upon completion of the study, deidentified data supporting the findings of the study will be made available from the corresponding author upon reasonable request, in accordance with institutional and privacy regulations.

## Appendix

Multimedia Appendix 1 SPIRIT checklist.

Multimedia Appendix 2 Peer review report by CASEM (Canadian Academy of Sport and Exercise Medicine) Research Committee.

## Abbreviations

6MWT: 6-Minute Walk Test
ABC: Activities-specific Balance Confidence
CONSORT: Consolidated Standards of Reporting Trials
ICanWALK: Improving Canadians’ Walking Aid Skills, Learning, and Knowledge
ICC: intraclass correlation
mHealth: mobile health
RCT: randomized controlled trial
SPIRIT: Standard Protocol Items Recommendations for Intervention Trials
T1: baseline
T2: after intervention
T3: 4-week follow-up
TUG: Timed up and Go
WAST: Walking Aids Skills Test
