# Supplementary material for: Exploring the Influence of a Novel App for Training and Evaluating Walking Aid Skills in Walking Aid Users: Protocol for a Pragmatic Single-Blind Randomized Controlled Trial
Source: JMIR Res Protoc. 2025 Dec 24;14:e71060. doi: 10.2196/71060 (PMC12780705; doi:10.2196/71060)
Supplement: Multimedia Appendix 2 [file resprot_v14i1e71060_app2.pdf]

WCB research Program
9925 - 107 Street
P.O. Box 2415
Edmonton, Alberta T5J 2S5
Tel: (780) 498-7799
Fax: (780) 498-7884
WCB website: www.wcb.ab.ca

July 11, 2024

Dr. Ranita Manocha
Division of Physical Medicine & Rehabilitation
University of Calgary
AC144, Special Services Building
Foothills Medical Centre
1403-29 Street NW
Calgary AB T2N 2T9

Dear Dr. Manocha:

Re: 2024 Full Proposal Submission – Pragmatic implementation of a walking aid skills training program to prevent secondary harms

---

Congratulations! The WCB Research Committee has approved your full proposal for the project entitled *Pragmatic implementation of a walking aid skills training program to prevent secondary harms*. The Committee was pleased to receive and evaluate your high-quality submission, however, due to a highly competitive year and budget constraints, is only able to **partially fund your project in the amount of $34,200.00**. Please be aware that you are welcome to submit a request for additional funding in subsequent years of this research via our annual research grants competition process. The research funding agreement will include a Term date representing the entire length of this research project, as presented in the proposal – **September 9, 2024 to August 31, 2026** – for ease of account management.

Research sponsorship is contingent upon:

- Acknowledgement of and adherence to the terms and conditions outlined in a research funding agreement (forthcoming);
- Maintenance of ethics approval throughout the project's duration;
- Submission of quarterly project updates. A template will be e-mailed to you each quarter for completion;
- Submission of a final report upon the completion of your project. The final report may be a publication in a peer-reviewed journal or a report prepared exclusively for WCB.

I will send your research contract to you or a member of your team to submit for legal review at the University of Calgary. I will work with the Research Services Office to ensure that the contract is fully executed and at that time, a cheque or EFT for $34,200.00 will be sent for your project. You will receive a copy of the documentation for your records.

If you have any questions or need further information, please do not hesitate to contact me via email at amy.procter@wcb.ab.ca.

WCB research Program
9925 - 107 Street
P.O. Box 2415
Edmonton, Alberta T5J 2S5
Tel: (780) 498-7799
Fax: (780) 498-7884
WCB website: www.wcb.ab.ca

Congratulations once again on your success; we truly appreciate your dedication to your research goals and look forward to working with you! All the very best with your project.

Sincerely,

Amy Procter MA
Research Program Coordinator
Workers' Compensation Board – Alberta
